# How to characterize a nonlinear elastic material? A review on nonlinear constitutive parameters in isotropic finite elasticity

**DOI:** 10.1098/rspa.2017.0607

**Published:** 2017-11-29

**Authors:** L. Angela Mihai, Alain Goriely

**Affiliations:** 1 School of Mathematics, Cardiff University, Senghennydd Road, Cardiff, CF24 4AG, UK; 2 Mathematical Institute, University of Oxford, Woodstock Road, Oxford, OX2 6GG, UK

**Keywords:** nonlinear elasticity, large strain, hyperelastic models, rubber, soft tissue, foams

## Abstract

The mechanical response of a homogeneous isotropic linearly elastic material can be fully characterized by two physical constants, the Young’s modulus and the Poisson’s ratio, which can be derived by simple tensile experiments. Any other linear elastic parameter can be obtained from these two constants. By contrast, the physical responses of nonlinear elastic materials are generally described by parameters which are scalar functions of the deformation, and their particular choice is not always clear. Here, we review in a unified theoretical framework several nonlinear constitutive parameters, including the stretch modulus, the shear modulus and the Poisson function, that are defined for homogeneous isotropic hyperelastic materials and are measurable under axial or shear experimental tests. These parameters represent changes in the material properties as the deformation progresses, and can be identified with their linear equivalent when the deformations are small. Universal relations between certain of these parameters are further established, and then used to quantify nonlinear elastic responses in several hyperelastic models for rubber, soft tissue and foams. The general parameters identified here can also be viewed as a flexible basis for coupling elastic responses in multi-scale processes, where an open challenge is the transfer of meaningful information between scales.

The task of the theorist is to bring order into the chaos of the phenomena of nature, to invent a language by which a class of these phenomena can be described efficiently and simply.

—Clifford Truesdell (1965) [1].

## 1. Introduction

An elastic body or material is *linear elastic* or *Hookean* if the force needed to extend or compress it by some distance is proportional to that distance [2]. The mechanical response of a homogeneous isotropic linearly elastic material is fully characterized by two physical constants that can be derived by simple experiments. For instance, a uniaxial tension or compression yields both the Young’s modulus and the Poisson ratio. Any other linear elastic parameter can then be obtained from these two constants [3]. The assumption that, under the small strain regime, materials are linearly elastic with possibly a geometrically nonlinear behaviour is successfully used in many engineering applications.

However, many modern applications and biological materials involve large strains, whereby the deformations are inherently nonlinear and the corresponding stresses depend on the underlying material properties. Biological and bioinspired materials are the subject of continuous intensive research efforts in biomedical applications, and can also be found in everyday life as well as in several industrial areas, e.g. microelectronics, aerospace, pharmaceutical and food processes. For these complex materials, reliable models supported by rigorous mechanical analysis are needed and can also open the way to new applications [4–12].

Here, we concentrate on the nonlinear elastic response of materials and do not discuss possible viscoelastic behaviours which may be relevant in many biological systems. In general, the mechanical responses of nonlinear elastic materials cannot be represented by constants but are described by parameters which are scalar functions of the deformation. The complexity of defining such functions comes from the fact that there are multiple ways to define strains and stresses in nonlinear deformations, giving rise to multiple nonlinear functions corresponding to the same linear parameter. Furthermore, the choice of these functions depends on how a particular experiment is conducted and how the experimental data are processed [13–18]. For an elastic material subject to large strains, the usual approach is to approximate directly the constants appearing in the mathematical model by employing numerical optimization techniques in order to minimize the residual between the stress–strain relation and the experimental data. Standard physical experiments are conducted mostly under uniaxial or biaxial loads [19–31], and less frequently, under simple or pure shear and torsional loading [25,28,32–34], while combined shear and axial, or torsion and axial, experiments are rarer [20,26,35,36].

When the geometries and boundary conditions of the deforming body are more complex, or application-specific, inverse finite-element modelling can be employed [37–40]. This involves the simulation of experiments whereby the material parameters are altered until the force–displacement responses in the simulations match those measured by the experiments [24,41,42]. For many practical applications, this can be very expensive computationally, especially when complex geometries and a very fine mesh are involved. In addition, as the modelling errors and the computational ones are undistinguishable, the model verification and validation processes are prohibitive [43–45]. Hence, the choice of one set of computed parameters versus another remains unclear [46]. Moreover, although under given forces, many isotropic elastic materials deform uniquely, for nonlinear hyperelastic materials, this is not always the case [47,48,49]. In practice, hyperelastic models containing fewer terms and constant coefficients, which can be altered more easily or related directly to the linear elastic constitutive parameters, are usually preferred even if their approximation of the experimental data is not the best [16,20,22,50–55]. This is further underpinned by the fact that, for more complex models, no particular physical interpretation can be attributed to every individual constituent, which may increase the risk of overfitting [56,57].

An alternative approach is to regard individual constants in a hyperelastic model as (non-unique) contributors to *general constitutive parameters that are explicit functions of the deformation* and convey nonlinear material properties that can be estimated directly from experimental measurements. In this review, we consider nonlinear constitutive parameters for homogeneous isotropic hyperelastic materials within the theoretical framework of finite elasticity, which in principle can provide a complete description of elastic responses in a solid material under loading [9,58–64]. In §2, we give a very short introduction to the finite elasticity theory of homogeneous isotropic hyperelastic materials relevant to our discussion. In §3, for an elastic material subject to triaxial stretch, we define and compare the nonlinear Poisson functions and the bulk and stretch moduli in terms of different strain and stress tensors. In §§4 and 5, for an elastic body subject to simple shear, or simple torsion, superposed on axial stretch, we define the associated nonlinear shear or torsion moduli, respectively, and relate them to the nonlinear stretch moduli via important universal relations. We recall that universal relations are equations that hold for every material in a specified class [65–67]. The key nonlinear parameters discussed here are summarized in tables 1–4. Note that, in the small strain limit, these parameters can be identified with the usual values from the linear elasticity theory. In §6, we illustrate with examples how the general constitutive parameters defined here can be employed to capture nonlinear elastic responses in different applications involving large strain deformations.

**Table 1.** Nonlinear Poisson functions for homogeneous isotropic hyperelastic materials subject to finite axial stretch (3.1), with stretch parameter *a*>0. In the small strain limit, these functions are equal to the Poisson’s ratio $\bar{\nu }$ from linear elasticity.

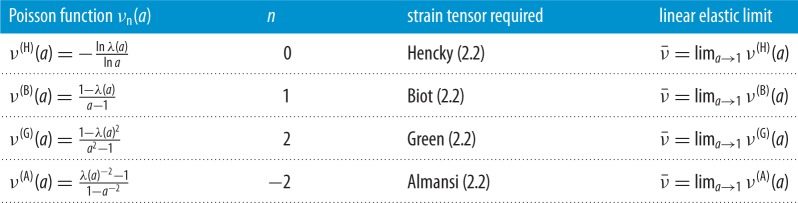

**Table 2.** Nonlinear stretch moduli for homogeneous isotropic hyperelastic materials subject to finite axial stretch (3.1), with stretch parameter *a*>0. In the small strain limit, these moduli are equal to the Young’s modulus $\bar{E}$ from linear elasticity.

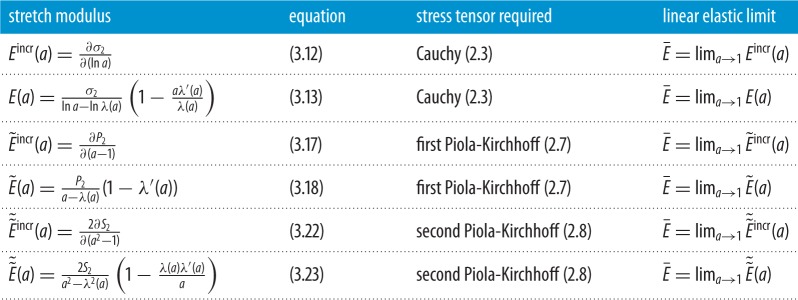

**Table 3.** Nonlinear shear moduli for homogeneous isotropic hyperelastic materials subject to simple shear superposed on finite axial stretch (4.1). In the small strain limit, these moduli are equal to the shear modulus $\bar{\mu }$ from linear elasticity.

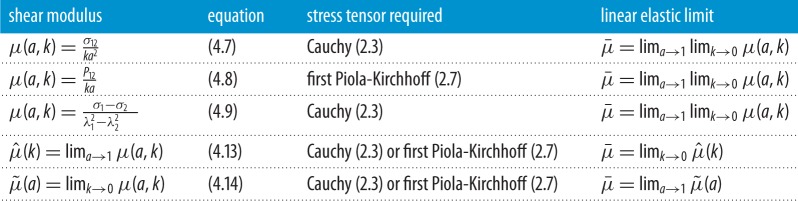

**Table 4.** Universal relation between nonlinear elastic parameters of homogeneous isotropic hyperelastic materials in the general case where the nonlinear Poisson’s ratio *ν*^(*H*)^(*a*)=*ν*_0_(*a*) defined by (3.7) changes with the deformation, and in the particular case when $\nu^{(H)}(a)=\bar{\nu }$ is constant.

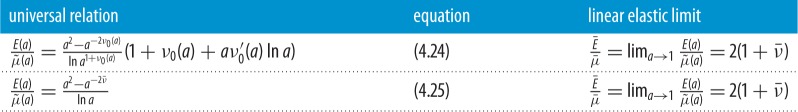

## 2. Nonlinear elastic deformations

We consider a continuous three-dimensional material body in a compact domain $\bar{\Omega }\subset R^{3}$ subject to a finite elastic deformation defined by the one-to-one, orientation-preserving transformation $\chi :\Omega \to R^{3}$. We denote by **X** the Lagrangian (reference, material) coordinates and by **x** the Eulerian (current, spatial) coordinates of a material point, respectively. The deformation gradient is **F**=∇*χ*=Grad **x**(**X**), with J=detF>0. The corresponding displacement field is defined as [9, p. 263] **u**(**X**)=**x**−**X**, and the displacement gradient is equal to ∇**u**=Grad **u**=**F**−**I**, where **I** is the identity tensor.

### (a) Strain tensors

To define the nonlinear strain tensors, we will make use of the *polar decomposition theorem* [9, p. 276], which states that: **F** has two unique multiplicative decompositions of the form **F**=**RU** and **F**=**VR**, where **U**=(**F**^T^**F**)^1/2^ and **V**=(**FF**^T^)^1/2^ are symmetric and positive definite, representing the right and left stretch tensors, respectively, and **R** is proper orthogonal (i.e. **R**^−1^=**R**^T^, with the superscript *T* denoting transpose, and detR=1), representing the rotation tensor. Of particular significance are the right Cauchy–Green tensor **C**=**U**^2^=**F**^T^**F** and the left Cauchy–Green tensor **B**=**V**^2^=**FF**^T^. As **V**=**RUR**^T^, the right and left stretch tensors **U** and **V** have the same eigenvalues {λ_*i*_}_*i*=1,2,3_, called the principal stretches. It follows that **B**=**V**^2^=**RU**^2^**R**^T^=**RCR**^T^, i.e. the right and left Cauchy–Green tensors have the same eigenvalues ${\left\{\lambda_{i}^{2}\right\}}_{i=1,2,3}$. The principal invariants of the Cauchy–Green tensors **B** and **C** are [68]

2.1 $$\begin{array}{rl} I_{1} & =\mathrm{tr}\;(\text{B})=\lambda_{1}^{2}+\lambda_{2}^{2}+\lambda_{3}^{2},\\ I_{2} & =\frac{1}{2}[{\left(\mathrm{tr}\;\text{B}t\right)}^{2}-\mathrm{tr}({\text{B}}^{2})]=\lambda_{1}^{2}\lambda_{2}^{2}+\lambda_{2}^{2}\lambda_{3}^{2}+\lambda_{3}^{2}\lambda_{1}^{2}\\ \text{and}\;I_{3} & =\det\;\text{B}=\lambda_{1}^{2}\lambda_{2}^{2}\lambda_{3}^{2}. \end{array}\}$$

From these basic kinematic quantities, we can define strain tensors. Here, we identify a one-parameter family of tensors combining both Lagrangian and Eulerian strain tensors [63, pp. 156,159]:

2.2 $${\text{e}}_{n}=\left\{ \begin{array}{ll} \frac{{\text{C}}^{n/2}-\text{I}}{n} & \text{if}\;n>0,\\ \ln\;{\text{C}}^{1/2} & \text{if}\;n=0,\\ \frac{{\text{B}}^{n/2}-\text{I}}{n} & \text{if}\;n<0. \end{array}\right.$$

Some of these tensors are routinely used, such as the *Hencky (logarithmic or true) strain tensor* [69] **e**^(H)^=**e**_0_, the *Biot strain tensor* [70] **e**^(B)^=**e**_1_, the *Green strain tensor* [63, pp. 89–90] **e**^(G)^=**e**_2_, the *Almansi strain tensor* [63, pp. 90–91] **e**^(A)^=**e**_−2_. The strain tensors **e**_*n*_ defined by (2.2) are independent of rotation, and for small elastic deformations, they are equivalent to the infinitesimal strain from the linear elastic theory $\bar{\text{e}}=(\nabla \text{u}+\nabla {\text{u}}^{T})/2$. Throughout this review, the bar over a scalar or a tensor is used to denote a value appearing in the theory of linear elasticity.

### (b) Stress tensors

We focus on homogeneous isotropic hyperelastic materials described by a strain-energy density function that depends only on the deformation gradient **F** and is identically zero at the unstressed state, i.e. W(I)=0. By the principle of objectivity, requiring that the strain-energy function is unaffected by a superimposed rigid-body deformation, which involves a change of position, and by the material symmetry, W can be expressed equivalently in terms of the principal invariants {*I*_1_,*I*_2_,*I*_3_}, or alternatively, in terms of the stretches {λ_1_,λ_2_,λ_3_}. To simplify the notation, we write the strain-energy function as W and infer its argument from the context. We define the following stress tensors:

— The *Cauchy stress tensor*, representing the force per unit area in the current configuration,
  2.3 $$\sigma =J^{-1}\frac{\partial W}{\partial \text{F}}{\text{F}}^{T}-p\text{I}=2J^{-1}\text{F}\frac{\partial W}{\partial \text{C}}{\text{F}}^{T}-p\text{I}=2J^{-1}\frac{\partial W}{\partial \text{B}}\text{B}-p\text{I},$$
  where *p*=0 for compressible materials and *J*=1 for incompressible materials. For incompressible materials, *p* is the Lagrange multiplier associated with the incompressibility constraint, commonly referred to as the arbitrary hydrostatic pressure [[9, p. 286, 17, 64, p. 74]. Note that the Cauchy stress tensor (2.3) is symmetric, i.e. ***σ***^T^=***σ***. For compressible materials, the Cauchy stress tensor (2.3) can be written equivalently as [64, p. 140]
  2.4 $$\sigma =2J^{-1}\;\left(\frac{\partial W}{\partial I_{1}}\frac{\partial I_{1}}{\partial \text{B}}+\frac{\partial W}{\partial I_{2}}\frac{\partial I_{2}}{\partial \text{B}}+\frac{\partial W}{\partial I_{3}}\frac{\partial I_{3}}{\partial \text{B}}\right)\text{B}=\beta_{0}\text{I}+\beta_{1}\text{B}+\beta_{-1}{\text{B}}^{-1},$$
  where the constitutive coefficients
  2.5 $$\beta_{0}=\frac{2}{\sqrt{I_{3}}}\left(I_{2}\frac{\partial W}{\partial I_{2}}+I_{3}\frac{\partial W}{\partial I_{3}}\right),\;\beta_{1}=\frac{2}{\sqrt{I_{3}}}\frac{\partial W}{\partial I_{1}}\;\mathrm{and}\;\beta_{-1}=-2\sqrt{I_{3}}\frac{\partial W}{\partial I_{2}}$$
  are scalar functions of the invariants (2.1) [64, p. 23]. Thus the Cauchy stress tensor ***σ*** and the left Cauchy–Green tensor **B** are coaxial, i.e. they have the same eigenvectors. When the material is incompressible, the stress tensor (2.3) is equal to
  2.6 $$\sigma =-p\;\text{I}+\beta_{1}\text{B}+\beta_{-1}{\text{B}}^{-1}.$$
— The *first Piola-Kirchhoff stress tensor*, representing the force per unit area in the reference configuration,
  2.7 $$\text{P}=J\sigma {\text{F}}^{-T}=\frac{\partial W}{\partial \text{F}}-p{\text{F}}^{-T},$$
  where *p*=0 for compressible materials and *J*=1 for incompressible materials. The stress tensor (2.7) is not symmetric in general.
— The *second Piola-Kirchhoff stress tensor*,
  2.8 $$\text{S}={\text{F}}^{-1}\text{P}=J{\text{F}}^{-1}\sigma {\text{F}}^{-T}=2\frac{\partial W}{\partial \text{C}}-p{\text{C}}^{-1},$$
  where *p*=0 for compressible materials and *J*=1 for incompressible materials. This stress tensor has no physical interpretation, but it is sometimes preferred, due to its symmetry, especially in computational approaches [37–39]. For compressible materials, the stress tensor (2.8) has the equivalent representation
  2.9 $$\text{S}=2\;\left(\frac{\partial W}{\partial I_{1}}\frac{\partial I_{1}}{\partial \text{C}}+\frac{\partial W}{\partial I_{2}}\frac{\partial I_{2}}{\partial \text{C}}+\frac{\partial W}{\partial I_{3}}\frac{\partial I_{3}}{\partial \text{C}}\right)=\gamma_{0}\text{I}+\gamma_{1}\text{C}+\gamma_{-1}{\text{C}}^{-1},$$
  where
  2.10 $$\gamma_{0}=2\;\left(\frac{\partial W}{\partial I_{1}}+I_{1}\frac{\partial W}{\partial I_{2}}\right),\;\gamma_{1}=-2\frac{\partial W}{\partial I_{2}}\;\mathrm{and}\;\gamma_{-1}=2I_{3}\frac{\partial W}{\partial I_{3}}$$
  are scalar functions of the principal invariants (2.1). Hence, the second Piola-Kirchhoff stress tensor **S** and the right Cauchy–Green tensor **C** are coaxial. When the material is incompressible, the stress tensor (2.8) is equal to
  2.11 $$\text{S}=\gamma_{0}\text{I}+\gamma_{1}\text{C}-p_{0}{\text{C}}^{-1},$$
  where *γ*_0_ and *γ*_1_ are given by (2.10) and *p*_0_ is the arbitrary hydrostatic pressure.

For the stress tensors (2.3), (2.7) and (2.8), the principal components (i.e. their principal eigenvalues) can be expressed in terms of derivatives of W with respect to the principal stretches (see appendix A where different explicit forms for the principal stress components are given).

### (c) Incremental elastic moduli

Assuming that the strain-energy function W is an analytic function of the strain tensor **e**, using Einstein’s notation convention that repeated indices represent summation, this function can be approximated as follows [39, p. 219]:

2.12 $$W\approx E_{0}+E_{ij}e_{ij}+\frac{1}{2}E_{ijkl}e_{ij}e_{kl},$$

where *E*_0_ is an arbitrary constant, {*E*_*ij*_}_*i*,*j*=1,2,3_ are elastic moduli of order 0 and {*E*_*ijkl*_}_*i*,*j*,*k*,*l*=1,2,3_ are elastic moduli of order 1 [63, p. 331]. The elastic moduli are defined to measure changes of the stress with the changes of strain. Such changes can be estimated, for example, by the following incremental fourth-order tensors:

— The gradient of the Cauchy stress tensor ***σ*** with respect to the logarithmic strain tensor $\ln\;{\text{B}}^{1/2}$,
  2.13 $${\text{E}}^{\text{incr}}=\frac{\partial \sigma }{\partial (\ln\;{\text{B}}^{1/2})}=\frac{\partial \sigma }{\partial (\ln\;\text{V})},$$
  with the components
  2.14 $$E_{ijkl}^{\text{incr}}=\frac{\partial \sigma_{ij}}{\partial (\ln\;V_{kl})},\;i,j,k,l=1,2,3.$$
— The gradient of the first Piola-Kirchhoff stress tensor **P** with respect to the deformation gradient **F**, or equivalently, the gradient of **P** with respect to the displacement gradient **F**−**I**,
  2.15 $${\text{E}}^{\text{incr}}=\frac{\partial \text{P}}{\partial \text{F}}=\frac{\partial \text{P}}{\partial (\text{F}-\text{I})},$$
  with the components
  2.16 $$E_{ijkl}^{\text{incr}}=\frac{\partial P_{ij}}{\partial F_{kl}}=\frac{\partial P_{ij}}{\partial (F_{kl}-\delta_{kl})},\;i,j,k,l=1,2,3.$$
  Then *E*^incr^_*ijkl*_>0 if the stress component *P*_*ij*_ increases as the strain component *F*_*kl*_−*δ*_*kl*_ increases, and *E*^incr^_*ijkl*_<0 if *P*_*ij*_ decreases as *F*_*kl*_−*δ*_*kl*_ increases. The fourth-order tensor (2.15) can be expressed equivalently as
  2.17 $${\text{E}}^{\text{incr}}=\frac{\partial^{2}W}{\partial {\text{F}}^{2}}=\frac{\partial^{2}W}{\partial {\left(\text{F}-\text{I}\right)}^{2}}.$$
  As, for the unstressed state, ∂W/∂(F−I)=P=0, by (2.12), we can write
  2.18 $$W\approx \frac{1}{2}E_{ijkl}^{\text{incr}}(F_{ij}-\delta_{ij})(F_{kl}-\delta_{kl}).$$
— The gradient of the second Piola-Kirchhoff stress tensor **S** with respect to the left Cauchy–Green tensor **C**, or equivalently, half of the gradient of **S** with respect to the Green strain tensor **e**^(G)^=(**C**−**I**)/2,
  2.19 $${\text{E}}^{\text{incr}}=\frac{\partial \text{S}}{\partial \text{C}}=\frac{\partial \text{S}}{\partial (\text{C}-\text{I})},$$
  with the components
  2.20 $$E_{ijkl}^{\text{incr}}=\frac{\partial S_{ij}}{\partial C_{kl}}=\frac{\partial S_{ij}}{\partial (C_{kl}-\delta_{kl})},\;i,j,k,l=1,2,3.$$
  Then *E*^incr^_*ijkl*_>0 if the stress component *S*_*ij*_ increases as the strain component (*C*_*kl*_−*δ*_*kl*_)/2 increases, and *E*^incr^_*ijkl*_<0 if *S*_*ij*_ decreases as (*C*_*kl*_−*δ*_*kl*_)/2 increases. The fourth-order tensor (2.19) takes the equivalent form
  2.21 $${\text{E}}^{\text{incr}}=2\frac{\partial^{2}W}{\partial {\text{C}}^{2}}.$$
  In this case, as, for the unstressed state, $\partial W/\partial {\text{e}}_{G}=\text{S}=\text{0}$, by (2.12), we can write
  2.22 $$W\approx \frac{1}{8}E_{ijkl}^{\text{incr}}(C_{ij}-\delta_{ij})(C_{kl}-\delta_{kl}).$$

The incremental elastic moduli (2.13), (2.15) and (2.19) can be calculated for any hyperelastic material for which the strain-energy function W is known, by using the definitions for the corresponding stress tensors in compressible or incompressible materials, respectively. When the strain-energy function is not known, assuming that the material is incompressible, these moduli can be approximated from a finite number of experimental measurements where the applied force is given. For compressible materials, suitable body forces may also need to be taken into account.

### (d) Adsciticious inequalities

For the behaviour of a hyperelastic material to be physically plausible, there are some universally accepted empirical requirements, which are constraints on the constitutive equations. These constraints take the form of inequalities and cannot be obtained from first principles, hence they are named *adscititious* or *empirical* [71–74, 9, p. 291, 64, pp. 153–171].

#### (i) Baker–Ericksen inequalities

For a hyperelastic body subject to uniaxial tension, the deformation is a simple extension in the direction of the tensile force if and only if the Baker–Ericksen (BE) inequalities stating that *the greater principal stress occurs in the direction of the greater principal stretch* hold [75,76]. The BE inequalities take the form

2.23 $$(\sigma_{i}-\sigma_{j})(\lambda_{i}-\lambda_{j})>0\;\text{if}\;\lambda_{i}\neq \lambda_{j},\;i,j=1,2,3,$$

where {λ_*i*_}_*i*=1,2,3_ and {*σ*_*i*_}_*i*=1,2,3_ are the principal stretches and the principal stresses, respectively, and ‘≥’ replaces the strict inequality ‘>’ in (2.23) if any two principal stretches are equal.

#### (ii) Pressure–compression inequalities

Another set of plausible constitutive constraints are the pressure–compression (PC) inequalities stating that *each principal stress is a pressure (compression) or a tension if the corresponding principal stretch is a contraction or an elongation (extension)* [64, p. 155]. In practice, either or both of the following ‘mean versions’ of the PC conditions are physically more realistic:

2.24 $$\sigma_{1}(\lambda_{1}-1)+\sigma_{2}(\lambda_{2}-1)+\sigma_{3}(\lambda_{3}-1)>0$$

or

2.25 $$\sigma_{1}\left(1-\frac{1}{\lambda_{1}}\right)+\sigma_{2}\left(1-\frac{1}{\lambda_{2}}\right)+\sigma_{3}\left(1-\frac{1}{\lambda_{3}}\right)>0,$$

if not all {λ_*i*_}_*i*=1,2,3_ are equal to 1.

**Remark 2.1**

The BE and the PC inequalities are verified by most elastic materials, as confirmed by experiments and experience. For a linear elastic material characterized by the shear and bulk moduli, the PC inequalities require that these moduli are both positive, while the BE inequalities only require that the shear modulus is positive. However, in finite elasticity in general, neither of these two sets of inequalities be implied by the other [64, pp. 155–159].

## 3. Experiment no. 1: simple tension or compression

For a hyperelastic body under uniaxial tension (or compression) acting in the second direction, the Cauchy stress takes the form

**Figure 1.**
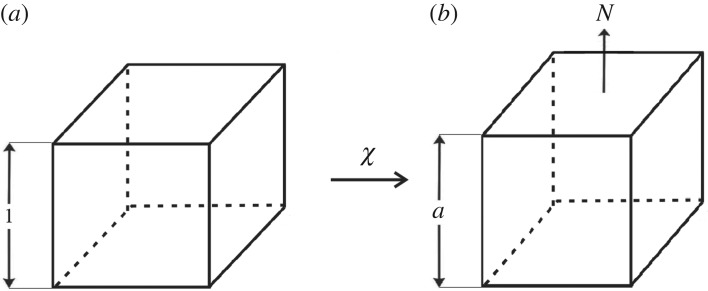
Cuboid (*a*) deformed by axial stretch (*b*) under the uniaxial load *N*.

3.1 σ=diag(0,N,0),

where diag(*a*,*b*,*c*) denotes the diagonal tensor with (*a*,*b*,*c*) on its diagonal and *N* is a non-zero constant. In this case, it is known that the corresponding deformation is a simple extension (or contraction) of the form (figure 1)

3.2 $$x_{1}=\lambda (a)X_{1},\;x_{2}=aX_{2}\;\mathrm{and}\;x_{3}=\lambda (a)X_{3},$$

where (*X*_1_,*X*_2_,*X*_3_) and (*x*_1_,*x*_2_,*x*_3_) are the Cartesian coordinates for the reference and current configuration, respectively, *a* is a positive constant representing the extension (or contraction) ratio and λ(*a*) is the stretch ratio in the orthogonal direction.

For the deformation (3.2), the deformation gradient

3.3 F=diag(λ(a),a,λ(a)),

is symmetric, hence the left and right Cauchy–Green tensors are equal,

3.4 $$\text{B}=\text{C}=\mathrm{diag}(\lambda {\left(a\right)}^{2},a^{2},\lambda {\left(a\right)}^{2}).$$

Then *a*>1 for *N*>0 (axial tension) and 0<*a*<1 for *N*<0 (axial compression) if and only if the BE inequalities (2.23) [64, p. 158] hold. In [77], it was shown that a simple tensile load, i.e. *N*>0 in (3.1), produces a simple extension, i.e. *a*>1 in (3.2), provided the following *empirical inequalities* hold: *β*_0_≤0, *β*_1_>0 and *β*_−1_≤0 [64, p. 158].

In the special case when this deformation is isochoric, i.e. det F=1, the orthogonal stretch takes the form $\lambda (a)=1/\sqrt{a}$. For this deformation, as *σ*_1_=*σ*_3_=0 and *σ*_2_=*N*, the BE inequalities (2.23) reduce to $\sigma_{2}(a-1/\sqrt{a})>0$, i.e. *σ*_2_>0 for *a*>1 and *σ*_2_<0 for *a*<1. Therefore, when the deformation (3.2) is isochoric, the PC inequalities (2.24) and (2.25) are equivalent to the BE inequalities (2.23). In particular, for incompressible hyperelastic materials in simple tension or compression, as any deformation is isochoric, the BE inequalities are equivalent to the PC inequalities.

Using (3.4), the strain tensors (2.2) are simply given by

3.5 $${\text{e}}_{n}=\text{diag}(e_{n}(\lambda (a)),e_{n}(a),e_{n}(\lambda (a))),$$

where, for any given stretch *x*>0, we define the *nonlinear strain* [78]

3.6 $$e_{n}(x)=\left\{ \begin{array}{ll} \ln(x) & \text{if}\;n=0,\\ \frac{x^{n}-1}{n} & \text{if}\;n\neq 0. \end{array}\right.$$

**Figure 2.**
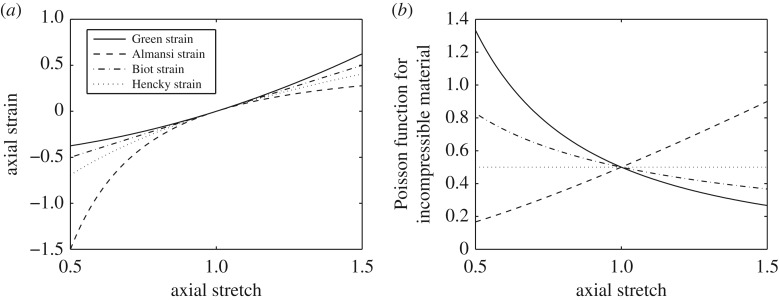
Comparison of different: (*a*) finite axial strains *e*_*n*_(*a*) versus axial stretch *a*; (*b*) nonlinear Poisson’s ratios for an incompressible material. Note that every axial strain increases with the increasing axial stretch, but only by using the Hencky (logarithmic) strain the corresponding Poisson function remains constant and equal to 0.5, capturing the characteristic property that the material volume remains fixed.

In figure 2*a*, we plot the values of different strain measures in the second direction as the stretch parameter *a* varies.

### (a) Nonlinear Poisson’s ratios

To introduce the nonlinear Poisson’s ratios, we consider an isotropic elastic material for which uniaxial loading causes a simple tension or compression (3.2). These deformations can be maintained in every homogeneous isotropic hyperelastic body by application of suitable traction [79–82]. Then the *nonlinear Poisson’s ratio* is defined as the negative quotient of the strain in an orthogonal direction to the strain in the direction of the applied force. Although, in practice, Poisson’s ratios are more often computed for small strains, this definition applies also in the case of large strains [83]. Whereas in the small strain regime the Poisson’s ratio is a constant, in finite strain, this ratio is a scalar function of the deformation. Moreover, for a nonlinear elastic material, the Poisson function can be expressed using different strains.

Using (3.5), we define a family of nonlinear Poisson functions as follows:

3.7 $$\nu_{n}(a)=-\frac{e_{n}(\lambda (a))}{e_{n}(a)}.$$

As before, we can specialize these functions for known strain tensors with the attached names: *Hencky form* with *ν*^(H)^(*a*)=*ν*_0_(*a*); *Biot form* with *ν*^(B)^(*a*)=*ν*_1_(*a*); *Green form* with *ν*^(G)^(*a*)=*ν*_2_(*a*) and *Almansi form* with *ν*^(A)^(*a*)=*ν*_−2_(*a*). In table 1, we summarize the nonlinear Poisson functions (3.7) for typical values of *n*.

The nonlinear Poisson’s ratios *ν*_*n*_(*a*) defined by (3.7) can be calculated directly from experimental measurements, without prior knowledge of the strain-energy function describing the material from which the elastic body undertaking the deformation is made. For infinitesimal deformations, i.e. when *a*→1, the Poisson’s ratios coincide with the Poisson’s ratio from the linear elastic theory,

3.8 $$\bar{\nu }=\lim_{a\to 1}\nu_{n}(a)=-\lim_{a\to 1}\lambda^{\prime }(a),$$

where λ′(*a*)=dλ(*a*)/d*a*. If a material is incompressible, then λ(*a*)=*a*^−1/2^ and the different nonlinear Poisson functions from table 1 are plotted in figure 2*b*. In particular, for *n*=0, λ(*a*)=*a*^−*ν*_0_(*a*)^, i.e. the Hencky form is the only Poisson function that remains constant and equal to $\nu_{0}(a)=\bar{\nu }=1/2$, capturing the characteristic conservation in the material volume.

For many materials, in the small strain regime, the Poisson’s ratio takes values between 0 and 0.5 [64, p. 154], but apparent Poisson’s ratios that are either negative or greater than 0.5 can also be obtained when large deformations occur. For anisotropic materials, different Poisson’s ratios may also be found as the material is extended or compressed in different directions. For example, negative Poisson’s ratios were reported in cork under non-radial (axial or transverse) compression [84], while Poisson’s ratios with values between 0.6 and 0.8 were measured in some woods where the primary strain was extensional in the radial direction and the secondary strain was compressive in the transverse direction [85]. An apparent Poisson’s ratio equal to 1 was also calculated for honeycomb structures with hexagonal cells under the small strain assumption [8].

### (b) Nonlinear bulk moduli

Volume changes can also be quantified by the *nonlinear bulk modulus*. Under finite triaxial deformation, we define this modulus as

3.9 $$\kappa =\frac{1}{3}\;\frac{\partial (\sigma_{1}+\sigma_{2}+\sigma_{3})}{\partial (J-1)},$$

where {*σ*_*i*_}_*i*=1,2,3_ are the axial stresses and *J*−1 is the volumetric strain.

For rubberlike materials, experiments which measure volume changes under finite uniaxial tension [[63, pp. 516–517], [86]] suggest that the bulk modulus remains constant and equal to the classical bulk modulus, i.e. $\kappa =\bar{\kappa }$. This seems to render the bulk modulus more attractive than the strain-dependent Poisson’s ratio when explicit material properties are sought experimentally. However, more experimental data exploring finite volume changes in elastic materials are needed.

In hydrostatic compression [87,88, 63, p. 519], nonlinear pressure versus volume responses of rubber materials were found. In his case, *σ*_1_=*σ*_2_=*σ*_3_=−*Jp*, where *p* is the hydrostatic pressure, and the bulk modulus (3.9) takes the simpler form [78]

3.10 $$\kappa =-J\frac{\partial p}{\partial J}-p.$$

Under small strain, the corresponding modulus is $\bar{\kappa }=-J\partial p/\partial J$. Volume change has also been observed under hydrostatic tension, but the deformation in this case is small before the elasticity limit is reached and cavitation occurs [63, p. 520].

### (c) Nonlinear stretch moduli

Another important quantifier of isotropic linear elasticity is the Young’s modulus. It is, therefore, important to define a nonlinear version of this parameter. For this purpose, we introduce the *nonlinear stretch modulus* to study the nonlinear elastic response of an isotropic hyperelastic material under the uniaxial tension or compression (3.1). The role of this elastic modulus is to reflect stiffening (or softening) in a material under increasing axial load. That is, when the axial stress increases as the axial deformation increases, there is an increase of the stretch modulus and the material stiffens, and if the axial stress decreases as the axial deformation increases, then there is a corresponding decrease in the stretch modulus as the material softens. We recall that, for uniaxial tension or compression, the first and third principal stretches are λ_2_=*a* and λ_1_=λ_3_=λ(*a*), respectively. As the stretch modulus depends on both a stress and a strain, there are multiple choices based on the particular stress and strain tensors considered. Here, we consider three typical moduli:

— For the Cauchy stress tensor, by subtracting the third from the second principal component given by (A 3), we obtain
  3.11 $$\sigma_{2}=\sigma_{2}-\sigma_{3}=(\ln\;a-\ln\;\lambda (a))\left(\zeta_{1}-\frac{\zeta_{-1}}{\ln\;a\;\ln\;\lambda (a)}\right).$$
  It follows that *σ*_2_ is proportional to ln a−ln λ(a), and similarly for incompressible materials, with λ(*a*)=*a*^−1/2^ if we subtract the third from the second principal component given by (A 14). Applying the general formula for the elastic moduli (2.14), we can define the *incremental stretch modulus* in terms of the logarithmic strain **e**_0_ as follows:
  3.12 $$E^{\mathrm{incr}}(a)=\frac{\partial \sigma_{2}}{\partial (\ln\;a)}=\frac{\partial \sigma_{2}}{\partial (\ln\;a-\ln\;\lambda (a))}\left(1-\frac{a\lambda^{\prime }(a)}{\lambda (a)}\right).$$
  Alternatively, as *σ*_2_ is proportional to ln a−ln λ(a), we can define a *nonlinear stretch modulus* of the form
  3.13 $$E(a)=\frac{\sigma_{2}}{\ln\;a-\ln\;\lambda (a)}\left(1-\frac{a\lambda^{\prime }(a)}{\lambda (a)}\right).$$
  For incompressible materials, where λ(*a*)=*a*^−1/2^, (3.13) simplifies to
  3.14 $$E(a)=\frac{\sigma_{2}(a)}{\ln\;a}.$$
  When *a*→1, i.e. for small axial strains, both the incremental modulus defined by (3.12), commonly known as the tangent modulus, and the nonlinear modulus given by (3.13), also known as the secant modulus, converge to the Young’s modulus from the linear elastic theory,
  3.15 $$\bar{E}=\lim_{a\to 1}E^{\mathrm{incr}}(a)=\lim_{a\to 1}E(a)=\lim_{a\to 1}\frac{\sigma_{2}(a)}{\ln\;a}.$$
— For the first Piola-Kirchhoff stress tensor, by subtracting the third from the second principal component given by (A 7), we find
  3.16 $$P_{2}=P_{2}-P_{3}=(a-\lambda (a))\left(\rho_{1}-\frac{\rho_{-1}}{a\lambda (a)}\right).$$
  Hence *P*_2_ is proportional to *a*−λ(*a*), and similarly for incompressible materials if we subtract the third from the second principal component given by (A 16). Applying the general formula for the elastic moduli (2.16), we define the *incremental stretch modulus* [37, p. 224]
  3.17 $${\tilde{E}}^{\mathrm{incr}}(a)=\frac{\partial P_{2}}{\partial (a-1)}=\frac{\partial P_{2}}{\partial (a-\lambda (a))}(1-\lambda^{\prime }(a)).$$
  In this case, as *P*_2_ is proportional to *a*−λ(*a*), we can also define the *nonlinear stretch modulus*
  3.18 $$\tilde{E}(a)=\frac{P_{2}}{a-\lambda (a)}(1-\lambda^{\prime }(a)).$$
  For incompressible materials, (3.18) takes the form
  3.19 $$\tilde{E}(a)=\frac{P_{2}}{a^{3/2}-1}\left(a^{1/2}+\frac{1}{2a}\right).$$
  When *a*→1, both elastic moduli (3.17) and (3.18) converge to the Young’s modulus
  3.20 $$\bar{E}=\lim_{a\to 1}E^{\mathrm{incr}}(a)=\lim_{a\to 1}\tilde{E}(a)=\lim_{a\to 1}\frac{\sigma_{2}}{\ln\;a}.$$
— For the second Piola-Kirchhoff stress tensor, subtracting the third from the second principal component given by (A 11) yields
  3.21 $$S_{2}=S_{2}-S_{3}=(a^{2}-\lambda^{2}(a))\left(\gamma_{1}-\frac{\gamma_{-1}}{a^{2}\lambda {\left(a\right)}^{2}}\right),$$
  i.e. *S*_2_ is proportional to *a*^2^−λ(*a*)^2^, and similarly for incompressible materials when we subtract the third from the second principal component given by (A 18). Then, using the formula for the elastic moduli (2.20), we define the following *incremental stretch modulus* in terms of the strain measure **e**_2_:
  3.22 $${\tilde{\tilde{E}}}^{\mathrm{incr}}(a)=\frac{2\partial S_{2}}{\partial (a^{2}-1)}=\frac{2\partial S_{2}}{\partial (a^{2}-\lambda {\left(a\right)}^{2})}\left(1-\frac{\lambda (a)\lambda^{\prime }(a)}{a}\right).$$
  Alternatively, as *S*_2_ is proportional to *a*^2^−λ(*a*)^2^, we can define the *nonlinear stretch modulus*
  3.23 $$\tilde{\tilde{E}}(a)=\frac{2S_{2}}{a^{2}-\lambda {\left(a\right)}^{2}}\left(1-\frac{\lambda (a)\lambda^{\prime }(a)}{a}\right).$$
  If the material is incompressible, then (3.23) becomes
  3.24 $$\tilde{\tilde{E}}(a)=\frac{2S_{2}}{a^{3}-1}\left(a+\frac{1}{2a^{2}}\right).$$
  When *a*→1, both moduli defined by (3.17) and (3.18), respectively, converge to the Young’s modulus
  3.25 $$\bar{E}=\lim_{a\to 1}E^{\mathrm{incr}}(a)=\lim_{a\to 1}\tilde{\tilde{E}}(a)=\lim_{a\to 1}\frac{\sigma_{2}}{\ln\;a}.$$

We summarize these nonlinear stretch moduli in table 2, and note that, when the strain-energy function is known, the incremental stretch moduli (3.12), (3.17) and (3.22) can be calculated from the definitions of the respective axial stresses (see §2b), but they are difficult to estimate accurately from a finite number of experimental measurements. However, when the strain-energy function is not known, the nonlinear stretch moduli (3.13), (3.18) and (3.23) can be obtained directly from experimental measurements where the axial force is prescribed. Moreover, while special assumptions regarding the strain-energy function are required in order for the incremental stretch moduli (3.12), (3.17) and (3.22) to be positive, the nonlinear stretch moduli (3.13), (3.18) and (3.23) are always positive if the PC inequalities (2.24) or (2.25) hold.

## 4. Experiment no. 2: simple shear superposed on axial tension

**Figure 3.**
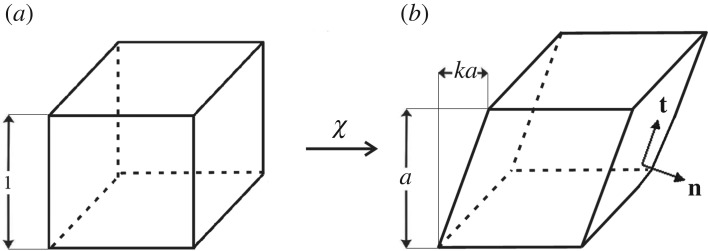
Cuboid (*a*) deformed by simple shear superposed on axial stretch (*b*), showing the unit normal and tangent vectors on an inclined face.

In isotropic linear elasticity, the Poisson’s ratio and Young’s modulus fully characterize a material. In particular, the response of a material under shear is given by its shear modulus $\bar{\mu }=\bar{E}/(2(1+\bar{\nu }))$. Yet, in nonlinear deformation the shear response cannot be simply obtained from the nonlinear Poisson’s ratio and the nonlinear stretch modulus. Therefore, we introduce the *nonlinear shear modulus* to study the nonlinear elastic response of an isotropic hyperelastic material subject to the following simple shear superposed on axial stretch [89] (figure 3),

4.1 $$x_{1}=\lambda (a)X_{1}+kaX_{2},\;x_{2}=aX_{2}\;\mathrm{and}\;x_{3}=\lambda (a)X_{3},$$

where (*X*_1_,*X*_2_,*X*_3_) and (*x*_1_,*x*_2_,*x*_3_) are the Cartesian coordinates for the reference and current configuration, respectively, and *a* and *k* are positive constants representing the axial stretch and the shear parameter, respectively. This deformation can be maintained in every homogeneous isotropic hyperelastic body by application of suitable traction [80,81,90].

For the deformation (4.1), the gradient tensor is

4.2 $$\text{F}=\left[\begin{array}{ccc} \lambda (a) & ka & 0\\ 0 & a & 0\\ 0 & 0 & \lambda (a) \end{array}\right]$$

and the left Cauchy–Green tensor is

4.3 $$\text{B}=\left[\begin{array}{ccc} \lambda {\left(a\right)}^{2}+k^{2}a^{2} & ka^{2} & 0\\ ka^{2} & a^{2} & 0\\ 0 & 0 & \lambda {\left(a\right)}^{2} \end{array}\right].$$

The corresponding principal stretches {λ_*i*_}_*i*=1,2,3_, such that ${\left\{\lambda_{i}^{2}\right\}}_{i=1,2,3}$ are the eigenvalues of the Cauchy–Green tensor (4.3), satisfy

4.4 $$\begin{array}{rl} \lambda_{1}^{2} & =\frac{\lambda {\left(a\right)}^{2}+a^{2}(1+k^{2})+\sqrt{{\left[\lambda {\left(a\right)}^{2}+a^{2}(1+k^{2})\right]}^{2}-4a^{2}\lambda {\left(a\right)}^{2}}}{2},\\ \lambda_{2}^{2} & =\frac{\lambda {\left(a\right)}^{2}+a^{2}(1+k^{2})-\sqrt{{\left[\lambda {\left(a\right)}^{2}+a^{2}(1+k^{2})\right]}^{2}-4a^{2}\lambda {\left(a\right)}^{2}}}{2}=a^{2}\lambda {\left(a\right)}^{2}\lambda_{1}^{-2}\\ \text{and}\;\lambda_{3}^{2} & =\lambda {\left(a\right)}^{2}, \end{array}\}$$

and the associated principal invariants (2.1) are equal to

4.5 $$\begin{array}{rl} I_{1} & =\lambda_{1}^{2}+\lambda_{2}^{2}+\lambda_{3}^{2}=k^{2}a^{2}+a^{2}+2\lambda {\left(a\right)}^{2},\\ I_{2} & =\lambda_{1}^{2}\lambda_{2}^{2}+\lambda_{2}^{2}\lambda_{3}^{2}+\lambda_{3}^{2}\lambda_{1}^{2}=k^{2}a^{2}\lambda {\left(a\right)}^{2}+2a^{2}\lambda {\left(a\right)}^{2}+\lambda {\left(a\right)}^{4}\\ \text{and}\;I_{3} & =\lambda_{1}^{2}\lambda_{2}^{2}\lambda_{3}^{2}=a^{2}\lambda {\left(a\right)}^{4}. \end{array}\}$$

By the representation (2.4), the non-zero components of the associated Cauchy stress are among the following components:

4.6 $$\begin{array}{rl} \sigma_{11} & =\sigma_{33}+k^{2}a^{2}\beta_{1},\\ \sigma_{12} & =ka^{2}\left(\beta_{1}-\frac{\beta_{-1}}{a^{2}\lambda {\left(a\right)}^{2}}\right),\\ \sigma_{22} & =\sigma_{33}+(a^{2}-\lambda {\left(a\right)}^{2})\left(\beta_{1}-\frac{\beta_{-1}}{a^{2}\lambda {\left(a\right)}^{2}}\right)+k^{2}\frac{\beta_{-1}}{\lambda {\left(a\right)}^{2}}\\ \text{and}\;\sigma_{33} & =\beta_{0}+\beta_{1}\lambda {\left(a\right)}^{2}+\frac{\beta_{-1}}{\lambda {\left(a\right)}^{2}}. \end{array}\}$$

Similar expressions are obtained for incompressible materials by specializing λ(*a*)=*a*^−1/2^ and by adding an arbitrary pressure to the diagonal terms of the Cauchy stress. For compressible and incompressible materials, the principal Cauchy stresses are given by (A 1)–(A 2) and (A 12)–(A 13), respectively.

### (a) Nonlinear shear moduli

By (4.6), the shear component of the Cauchy stress tensor, *σ*_12_, is proportional to *ka*^2^. In this case, a *nonlinear shear modulus* can be defined as follows:

4.7 $$\mu (a,k)=\frac{\sigma_{12}}{ka^{2}}=\beta_{1}-\frac{\beta_{-1}}{a^{2}\lambda {\left(a\right)}^{2}}.$$

For incompressible materials, as the shear component *P*_12_=*σ*_12_/*a* of the first Piola-Kirchhoff stress tensor (2.7) is proportional to the shear strain *ka*, the nonlinear shear modulus (4.7) is equal to

4.8 $$\mu (a,k)=\frac{P_{12}}{ka}=\frac{\sigma_{12}}{ka^{2}}=\beta_{1}-\frac{\beta_{-1}}{a}.$$

This modulus is independent of the Lagrange multiplier *p*, and can be estimated directly from experimental measurements if the shear force is known.

For both compressible and incompressible materials, by the representations (A 1)–(A 2) and (A 12)–(A 13) of the principal Cauchy stresses, respectively, the nonlinear shear modulus defined above can be written equivalently as

4.9 $$\mu (a,k)=\frac{\sigma_{1}-\sigma_{2}}{\lambda_{1}^{2}-\lambda_{2}^{2}}.$$

Hence, the nonlinear shear modulus (4.9) is positive if the BE inequalities (2.23) hold. Also, for a cuboid deformed by simple shear superposed on axial stretch (4.1), in the plane of shear, the unit normal and tangent vectors on the inclined faces are, respectively (figure 3),

4.10 $$\text{n}=\pm \frac{1}{\sqrt{1+k^{2}}}\left[\begin{array}{c} 1\\ -k\\ 0 \end{array}\right],\;\text{t}=\pm \frac{1}{\sqrt{1+k^{2}}}\left[\begin{array}{c} k\\ 1\\ 0 \end{array}\right]$$

and the tangent components of the Cauchy stress and left Cauchy–Green tensor are, respectively,

4.11 $$\sigma_{t}={\text{t}}^{T}\sigma \text{n}=\frac{k\lambda {\left(a\right)}^{2}}{1+k^{2}}\left(\beta_{1}-\frac{\beta_{-1}}{a^{2}\lambda {\left(a\right)}^{2}}\right)\;\mathrm{and}\;B_{t}={\text{t}}^{T}\text{B}\text{n}=\frac{k\lambda {\left(a\right)}^{2}}{1+k^{2}}.$$

Then (4.7) is equivalent to

4.12 $$\mu (a,k)=\frac{\sigma_{t}}{B_{t}}=\beta_{1}-\frac{\beta_{-1}}{a^{2}\lambda {\left(a\right)}^{2}}.$$

When *a*→1, i.e. for simple shear superposed on infinitesimal axial stretch, the nonlinear shear modulus given by (4.7) converges to the nonlinear shear modulus for simple shear [64, pp. 174–175],

4.13 $$\hat{\mu }(k)=\lim_{a\to 1}\mu (a,k)={\hat{\beta }}_{1}-{\hat{\beta }}_{-1},$$

where ${\hat{\beta }}_{1}=\lim_{a\to 1}\beta_{1}$ and ${\hat{\beta }}_{-1}=\lim_{a\to 1}\beta_{-1}$.

When *k*→0, i.e. for infinitesimal simple shear superposed on finite axial stretch, the nonlinear shear modulus given by (4.7) converges to

4.14 $$\tilde{\mu }(a)=\lim_{k\to 0}\mu (a,k)={\tilde{\beta }}_{1}-\frac{{\tilde{\beta }}_{-1}}{a^{2}\lambda {\left(a\right)}^{2}},$$

where ${\tilde{\beta }}_{1}=\lim_{k\to 0}\beta_{1}$ and ${\tilde{\beta }}_{-1}=\lim_{k\to 0}\beta_{-1}$. For incompressible materials,

4.15 $$\tilde{\mu }(a)={\tilde{\beta }}_{1}-\frac{{\tilde{\beta }}_{-1}}{a}.$$

We summarize the nonlinear shear moduli defined here in table 3. Note that, when *a*→1 and *k*→0, these moduli converge to the linear shear modulus of the infinitesimal theory [64, p. 179], i.e.

4.16 $$\bar{\mu }=\lim_{a\to 1}\lim_{k\to 0}\mu (a,k)=\lim_{k\to 0}\hat{\mu }(k)=\lim_{a\to 1}\tilde{\mu }(a)={\bar{\beta }}_{1}-{\bar{\beta }}_{-1},$$

where ${\bar{\beta }}_{1}=\lim_{a\to 1}\lim_{k\to 0}\beta_{1}$ and ${\bar{\beta }}_{-1}=\lim_{a\to 1}\lim_{k\to 0}\beta_{-1}$.

**Remark 4.1**

For simple shear, i.e. when *a*=1, the shear modulus (4.7) is in agreement with the generalized shear modulus defined by Truesdell & Noll [64, pp. 174–175], and also with the shear modulus defined by Moon & Truesdell [91]. However, for simple shear superposed on axial stretch, with *a*≠1, the shear modulus (4.7) differs by a factor *a*^2^ from the shear modulus in [91]. Nevertheless, for the nonlinear shear modulus defined here, the equivalent form (4.9) is valid for any *a*>0, including *a*=1 as in the simple shear case [64, p. 175].

### (b) Poynting modulus in shear

We recall that the (positive or negative) *Poynting effect* is a large strain effect observed when an elastic cube is sheared between two plates and stress is developed in the direction normal to the sheared faces, or when a cylinder is subjected to torsion and the axial length changes [28,32,91–97]. This effect naturally captures the coupling between normal and shear deformations when an elastic cube is sheared, and between axial and torsion deformations when a cylinder is twisted.

When an incompressible cube which is free on its outer surface is subject to simple shear, it exhibits an axial stretch proportional to the square of the shear,

4.17 $$|a-1|=|a(k)-1|=\mu_{P}k^{2}a^{2},$$

where the parameter *μ*_*P*_ is a positive constant. When *a*>1, the classical Poynting effect occurs, and if *a*<1, then the negative Poynting effect is observed. To estimate the value of *μ*_*P*_ in (4.17), identified here as the *Poynting modulus*, assuming *σ*_33_=0 in (4.6), as λ(*a*)=*a*^−1/2^, the normal force is equal to

4.18 $$N(a,k)=\sigma_{22}=\left(a^{2}-\frac{1}{a}\right)\left(\beta_{1}-\frac{\beta_{-1}}{a}\right)+k^{2}a^{2}\frac{\beta_{-1}}{a}.$$

Then, taking *N*(*a*,*k*)=0 in (4.18) provides an equation for the axial stretch *a* corresponding to the amount of shear *ka*. By (4.17) and (4.18), noting that *a*→1 as *k*→0, we obtain

4.19 $$\lim_{k\to 0}\frac{|a^{2}-1/a|}{k^{2}a^{2}}=\lim_{k\to 0}\left|\frac{{\tilde{\beta }}_{-1}/a}{{\tilde{\beta }}_{1}-{\tilde{\beta }}_{-1}/a}\right|=\frac{|{\bar{\beta }}_{-1}|}{{\bar{\beta }}_{1}-{\bar{\beta }}_{-1}}$$

and by (4.17) and (4.19),

4.20 $$\mu_{P}=\lim_{k\to 0}\frac{|a-1|}{k^{2}a^{2}}=\frac{1}{3}\lim_{k\to 0}\frac{|a^{3}-1|}{k^{2}a^{2}}=\frac{1}{3}\lim_{k\to 0}\frac{|a^{2}-1/a|}{k^{2}a^{2}}=\frac{|{\bar{\beta }}_{-1}|}{3({\bar{\beta }}_{1}-{\bar{\beta }}_{-1})}.$$

**Remark 4.2**

By (4.20), if *β*_−1_=0, then the Poynting modulus vanishes, meaning that the Poynting effect is not observed. When *β*_−1_<0, the classical Poynting effect occurs, and if *β*_−1_>0, then the negative Poynting effect is obtained. In [93,94], it was shown that positive or negative Poynting effects are possible if the following generalized empirical inequalities are assumed: *β*_0_≤0 and *β*_1_>0, without any constraint on *β*_−1_.

### (c) Universal relations between nonlinear shear and stretch moduli

For a unit cube of unconstrained material subject to simple shear superposed on finite axial stretch (4.1), when *σ*_33_=0 in (4.6), the normal force is equal to

4.21 $$N(a,k)=\sigma_{22}=(a^{2}-\lambda {\left(a\right)}^{2})\left(\beta_{1}-\frac{\beta_{-1}}{a^{2}\lambda {\left(a\right)}^{2}}\right)+k^{2}\frac{\beta_{-1}}{\lambda {\left(a\right)}^{2}}.$$

Taking the limit of infinitesimal shear, we obtain

4.22 $$\tilde{N}(a)=\lim_{k\to 0}N(a,k)=(a^{2}-\lambda {\left(a\right)}^{2})\left({\tilde{\beta }}_{1}-\frac{{\tilde{\beta }}_{-1}}{a^{2}\lambda {\left(a\right)}^{2}}\right)$$

and by (4.14),

4.23 $$\frac{\tilde{N}(a)}{\tilde{\mu }(a)}=\lim_{k\to 0}\frac{N(a,k)}{\mu (a,k)}=a^{2}-\lambda {\left(a\right)}^{2}.$$

Therefore, as the axial stretch *a*>1 increases, the magnitude of the normal force $\tilde{N}$ relative to the shear modulus $\tilde{\mu }$ increases. This is a *universal relation*, i.e. it holds independently of the material responses *β*_1_ and *β*_−1_. Recalling that, under infinitesimal simple shear, no Poynting effect is observed [93,94], i.e. the resulting normal force is zero, the following universal relation holds between the nonlinear shear modulus in the small shear limit (4.14) and the nonlinear stretch modulus (3.13) for the axial stretch *a* under the axial force $\tilde{N}(a)$:

4.24 $$\frac{E(a)}{\tilde{\mu }(a)}=\frac{\tilde{N}(a)}{\tilde{\mu }(a)}\frac{1}{\ln\;a-\ln\;\lambda (a)}\left(1-\frac{a\lambda^{\prime }(a)}{\lambda (a)}\right)=\frac{a^{2}-a^{-2\nu_{0}(a)}}{\ln\;a^{1+\nu_{0}(a)}}\left(1+\nu_{0}(a)+a\nu_{0}^{\prime }(a)\;\ln\;a\right).$$

If the Poisson’s ratio defined by *ν*^(H)^=*ν*_0_ is constant, $\nu_{0}=\bar{\nu }$, then $\lambda (a)=a^{-\nu_{0}}=a^{-\bar{\nu }}$ and the universal relation (4.24) becomes

4.25 $$\frac{E(a)}{\tilde{\mu }(a)}=\frac{\tilde{N}(a)}{\tilde{\mu }(a)\ln\;a}=\frac{a^{2}-a^{-2\bar{\nu }}}{\ln\;a}.$$

In particular, for incompressible materials, where $\bar{\nu }=1/2$,

4.26 $$\frac{E(a)}{\tilde{\mu }(a)}=\frac{\tilde{N}(a)}{\tilde{\mu }(a)\ln\;a}=\frac{a^{2}-a^{-1}}{\ln\;a}.$$

In the linear elastic limit, where *a*→1, the classical relation between the Young’s modulus and the linear shear modulus is recovered, i.e.

4.27 $$\frac{\bar{E}}{\bar{\mu }}=\lim_{a\to 1}\frac{E(a)}{\tilde{\mu }(a)}=2(1+\bar{\nu }).$$

For incompressible materials, $\bar{E}/\bar{\mu }=3$. The universal relations (4.24) and (4.25) and their linear elastic limits are summarized in table 4. These relations will be employed in the calibration of hyperelastic models to experimental data for rubberlike material in §6.

## 5. Experiment no. 3: simple torsion superposed on axial tension

**Figure 4.**
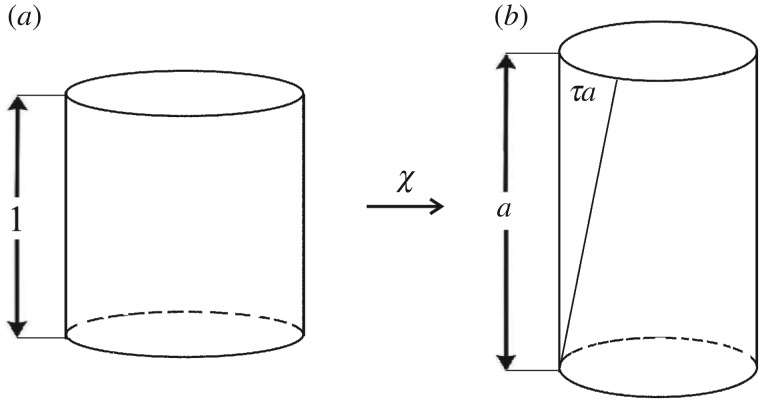
Circular cylinder (*a*) deformed by simple torsion superposed on axial stretch (*b*).

In this section, nonlinear elastic moduli are obtained under certain non-homogeneous finite deformations, which are controllable for all incompressible elastic solids in the absence of body forces. Generalizations of these deformations are also possible for specific isotropic compressible materials [98]. A circular cylinder of incompressible isotropic hyperelastic material occupying the domain (*R*,*Θ*,*Z*)∈[0,*R*_0_]×[0,2*π*]×[−*Z*_0_,*Z*_0_], where *R*_0_ and *Z*_0_ are positive constants, is deformed by the simple torsion superposed on axial stretch [64, pp. 189–191] (figure 4)

5.1 $$r=\frac{R}{\sqrt{a}},\;\theta =\Theta +\tau aZ,\;z=aZ,$$

where (*R*,*Θ*,*Z*) and (*r*,*θ*,*z*) are the cylindrical coordinates for the reference and the current configuration, respectively.

For this deformation, the deformation gradient is

5.2 $$\text{F}=\left[\begin{array}{ccc} \frac{\partial r}{\partial R} & 0 & 0\\ 0 & \left(\frac{r}{R}\right)\frac{\partial \theta }{\partial \Theta } & \frac{r\partial \theta }{\partial Z}\\ 0 & 0 & \frac{\partial z}{\partial Z} \end{array}\right]=\left[\begin{array}{ccc} \frac{1}{\sqrt{a}} & 0 & 0\\ 0 & \frac{1}{\sqrt{a}} & r\tau a\\ 0 & 0 & a \end{array}\right],$$

where *a* and *τ* are positive constants representing the axial stretch and the torsion parameter, respectively, and the left Cauchy–Green tensor is

5.3 $$\text{B}=\left[\begin{array}{ccc} \frac{1}{a} & 0 & 0\\ 0 & \frac{1}{a}+r^{2}\tau^{2}a^{2} & r\tau a^{2}\\ 0 & r\tau a^{2} & a^{2} \end{array}\right].$$

By (2.6), the non-zero components of the Cauchy stress tensor are among the following components:

5.4 $$\begin{array}{rl} \sigma_{rr} & =-p+\frac{\beta_{1}}{a}+\beta_{-1}a,\\ \sigma_{\theta \theta } & =\sigma_{rr}+\beta_{1}r^{2}\tau^{2}a^{2},\\ \sigma_{\theta z} & =r\tau a^{2}\left(\beta_{1}-\frac{\beta_{-1}}{a}\right)\\ \text{and}\;\sigma_{zz} & =\sigma_{rr}+\left(a^{2}-\frac{1}{a}\right)\left(\beta_{1}-\frac{\beta_{-1}}{a}\right)+r^{2}\tau^{2}a^{2}\frac{\beta_{-1}}{a}, \end{array}\}$$

where *p* depends only on *r*.

### (a) Nonlinear torsion moduli

The classical torsion modulus is measured as the ratio between the torque and the twist. For the deformation (5.1), if *B*_*rr*_<1 and *σ*_*rr*_=−*p*_0_≤0 at the external surface *r*=*r*_0_, then at this surface, the torque is equal to [64, pp. 190–191]

5.5 $$T(a,\tau )=2\pi \int_{0}^{r_{0}}\sigma_{\theta z}r^{2}\;dr=2\pi \tau a^{2}\int_{0}^{r_{0}}\left(\beta_{1}-\frac{\beta_{-1}}{a}\right)r^{3}\;dr.$$

The resultant normal force is [64, p. 191]

5.6 $$\begin{array}{rl} N(a,\tau ) & =2\pi \int_{0}^{r_{0}}\sigma_{zz}r\;dr\\ & =2\pi \int_{0}^{r_{0}}(\sigma_{zz}-\sigma_{rr})r\;dr+2\pi \int_{0}^{r_{0}}\sigma_{rr}r\;dr\\ & =-\pi p_{0}r_{0}^{2}+2\pi \left(a^{2}-\frac{1}{a}\right)\int_{0}^{r_{0}}\left(\beta_{1}-\frac{\beta_{-1}}{a}\right)r\;dr-\pi \tau^{2}a^{2}\int_{0}^{r_{0}}\left(\beta_{1}-\frac{2\beta_{-1}}{a}\right)r^{3}\;dr. \end{array}$$

As the torque is proportional to the twist, we define the nonlinear torsion modulus as the ratio between the torque *T* given by (5.5) and the amount of twist *τa*,

5.7 $$\mu_{T}(a,\tau )=\frac{T}{\tau a}=2\pi a\int_{0}^{r_{0}}\left(\beta_{1}-\frac{\beta_{-1}}{a}\right)r^{3}\;dr=\frac{2\pi }{a}\int_{0}^{R_{0}}\left(\beta_{1}-\frac{\beta_{-1}}{a}\right)R^{3}\;dR.$$

Note that this modulus increases as the radius *R*_0_ of the (undeformed) cylinder increases. When *a*→1, i.e. for simple torsion superposed on infinitesimal axial stretch, the modulus defined by (5.7) converges to the torsion modulus for simple torsion [64, p. 192],

5.8 $${\hat{\mu }}_{T}(\tau )=\lim_{a\to 1}\frac{T}{\tau }=\frac{\pi r_{0}^{4}}{2}({\hat{\beta }}_{1}-{\hat{\beta }}_{-1})=\frac{\pi R_{0}^{4}}{2}({\hat{\beta }}_{1}-{\hat{\beta }}_{-1}),$$

where ${\hat{\beta }}_{1}=\lim_{a\to 1}\beta_{1}$ and ${\hat{\beta }}_{-1}=\lim_{a\to 1}\beta_{-1}$. When *τ*→0, i.e. for infinitesimal torsion superposed on finite axial stretch, the modulus given by (5.7) converges to

5.9 $${\tilde{\mu }}_{T}(a)=\lim_{\tau \to 0}\mu_{T}(a,\tau )=\lim_{\tau \to 0}\frac{T}{\tau a}=\frac{\pi ar_{0}^{4}}{2}\left({\tilde{\beta }}_{1}-\frac{{\tilde{\beta }}_{-1}}{a}\right)=\frac{\pi R_{0}^{4}}{2a}\left({\tilde{\beta }}_{1}-\frac{{\tilde{\beta }}_{-1}}{a}\right),$$

where ${\tilde{\beta }}_{1}=\lim_{\tau \to 0}\beta_{1}$ and ${\tilde{\beta }}_{-1}=\lim_{\tau \to 0}\beta_{-1}$. If *τ*→0 and *a*→1, then these moduli converge to the linear elastic modulus

5.10 $${\bar{\mu }}_{T}=\lim_{a\to 1}\lim_{\tau \to 0}\mu_{T}(\tau ,a)=\lim_{a\to 1}{\hat{\mu }}_{T}(\tau )=\lim_{\tau \to 0}{\tilde{\mu }}_{T}(a)=\frac{\pi r_{0}^{4}}{2}({\bar{\beta }}_{1}-{\bar{\beta }}_{-1})=\frac{\pi R_{0}^{4}}{2}({\bar{\beta }}_{1}-{\bar{\beta }}_{-1}),$$

where ${\bar{\beta }}_{1}=\lim_{a\to 1}\lim_{\tau \to 0}\beta_{1}$ and ${\bar{\beta }}_{-1}=\lim_{a\to 1}\lim_{\tau \to 0}\beta_{-1}$.

### (b) Poynting modulus in torsion

The Poynting effect for an incompressible cylinder under torsion consists of an axial stretch proportional to the square of the twist, i.e.

5.11 $$|a-1|=|a(\tau )-1|=\mu_{P}\tau^{2}a^{2},$$

where the positive constant *μ*_*P*_ is identified as the *Poynting modulus* [64, p. 193]. To find the value of this modulus, we note that setting $N(a,\tau )=-p_{0}\pi r_{0}^{2}$ in (5.6) provides an equation for the axial stretch *a* corresponding to the amount of twist *τa*. In this case, by (5.6), *a*→1 as *τ*→0, and

5.12 $$\lim_{\tau \to 0}\frac{|a^{2}-1/a|}{\tau^{2}a^{2}}=\lim_{\tau \to 0}\left|\frac{\int_{0}^{r_{0}}({\tilde{\beta }}_{1}-2{\tilde{\beta }}_{-1}/a)r^{3}\;dr}{2\int_{0}^{r_{0}}({\tilde{\beta }}_{1}-{\tilde{\beta }}_{-1}/a)r\;dr}\right|=\frac{R_{0}^{2}}{4}\left|1-\frac{{\bar{\beta }}_{-1}}{{\bar{\beta }}_{1}-{\bar{\beta }}_{-1}}\right|.$$

Then, by (5.11), as *a*=*a*(*τ*)→1 as *τ*→0, we obtain

5.13 $$\mu_{P}=\lim_{\tau \to 0}\frac{|a-1|}{\tau^{2}a^{2}}=\frac{1}{3}\lim_{\tau \to 0}\frac{|a^{3}-1|}{\tau^{2}a^{2}}=\frac{1}{3}\lim_{\tau \to 0}\frac{|a^{2}-1/a|}{\tau^{2}a^{2}}=\frac{R_{0}^{2}}{12}\left|1-\frac{{\bar{\beta }}_{-1}}{{\bar{\beta }}_{1}-{\bar{\beta }}_{-1}}\right|,$$

where the last equality follows from (5.12). Hence, the Poynting modulus (5.13) increases as the radius *R*_0_ of the (undeformed) cylinder increases.

## 6. Examples and applications

Every linear elastic material can be characterized by two physical constants, which may be found from simple uniaxial tension or compression experiments. By contrast, the mechanical responses of nonlinear elastic materials cannot be represented by constants but are generally described by parameters which are functions of the deformation. To be effective in estimating elastic material behaviours, these parameters must satisfy certain criteria:

(i) For the nonlinear parameters to be generally applicable, they must be obtainable for all materials in a class, such as, for example, all compressible or incompressible homogeneous isotropic hyperelastic materials.
(ii) Ideally, nonlinear elastic parameters should be measured and calibrated under multiaxial deformations, which, in principle, are closer to real physical situations.
(iii) For mechanical consistency with the linear elasticity theory, these parameters must be equal to the corresponding linear elastic ones under small strains.

An important parameter that satisfies the aforementioned criteria is the nonlinear shear modulus *μ*(*a*,*k*) defined by (4.9). In table 5, for 12 popular incompressible isotropic hyperelastic models, we provide the explicit forms for this nonlinear shear modulus, its limit in the case of small shear superposed on finite axial stretch, $\tilde{\mu }(a)=\lim_{k\to 0}\mu (a,k)$ given by (4.15), and its linear elastic limit $\bar{\mu }=\lim_{a\to 1}\lim_{k\to 0}\mu (a,k)=\lim_{a\to 1}\tilde{\mu }(a)$ given by (4.16). For each model, the nonlinear shear modulus under simple shear, $\hat{\mu }(k)=\lim_{a\to 1}\mu (a,k)$ defined by (4.13), can also be derived, while the nonlinear stretch modulus *E*(*a*) can be inferred from the universal relation (4.26). The table clearly shows that although some materials have the same linear shear modulus (e.g. $\bar{\mu }=c_{1}$ for neo-Hookean, Yeoh, Fung and Gent models; $\bar{\mu }=c_{1}+c_{2}$ for Mooney-Rivlin, Carroll, Gent-Thomas and Gent–Gent models; $\bar{\mu }=\sum_{p=1}^{n}c_{p}$ for Ogden and Lopez-Pamies models), the nonlinear shear moduli are specific to each model, distinguishing them with respect to their elastic responses under large strains.

**Table 5.** Explicit forms of the shear moduli *μ*(*a*,*k*) of (4.9), $\tilde{\mu }(a)=\lim_{k\to 0}\mu (a,k)$ of (4.15), and $\bar{\mu }=\lim_{a\to 1}\tilde{\mu }(a)$ of (4.16) for selected incompressible isotropic hyperelastic models. For the shear moduli of these incompressible materials, the principal stretches are given by (4.4) with λ(*a*)=*a*^−1/2^.

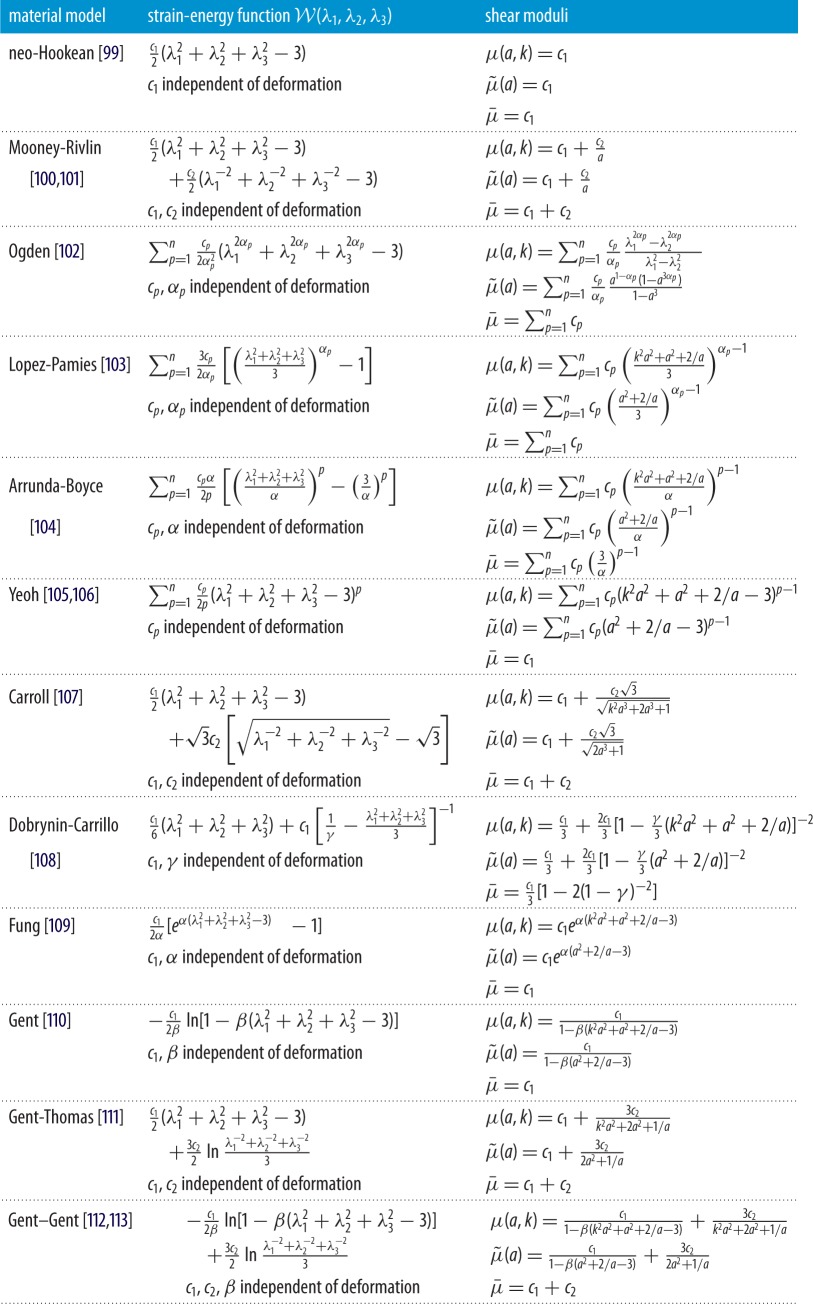

Equipped with these parameters, we then proceed to illustrate their application to certain materials, such as rubber, soft tissues and foams. The theoretical and practical challenges raised when modelling rubber elasticity are discussed in [17], which concludes with the open remark that for a theory to be helpful in explaining the elastic responses of this material, it should take into account its properties *‘not only in simple extension and compression, but also in other types of strain’*. Clearly, this is valid also for other elastic materials operating in large strain, and in particular, for soft tissues and foams, which are of growing research interest due to the great diversity of their nonlinear mechanical responses under loads.

In this context, the universal relations between the nonlinear shear and stretch moduli incorporate valuable information from both shear and axial deformations, and hence can be employed to quantify elastic responses in multiaxial deformation. Another nonlinear parameter that naturally captures the coupling between large axial and shear deformations is the Poynting modulus, which has received less attention in practical applications to date.

### (a) Rubber

The first experimental results on natural gum rubber were reported by Rivlin & Saunders [114] (see also [115, Ch. 5, 64, pp. 181–182]). In [116], fourteen hyperelastic models are also surveyed and their performance compared with Treloar’s elastomer data, which are provided as well (see also the models and discussions in [87,117–120]). For rubberlike materials under large tension, in [52], several hyperelastic models were systematically calibrated to experimental data measuring the tensile stress, and the corresponding values of second- and third-order elasticity constants were calculated. Recognizing the need for more information which is not represented by the stress–strain curve, the so-called *Mooney plot* has been proposed to capture additional behaviours in the calibrated models. The auxiliary function behind the Mooney plot takes the general form $g(z)=\partial W/\partial I_{1}+z\partial W/\partial I_{2}$, where *z*=1/*a* and *a* is the extension ratio [121]. In particular, for the Mooney-Rivlin model listed in table 5, the linear form *g*(*z*)=*c*_1_+*c*_2_*z* is obtained. It is interesting to note that although the value of this function is the same as that of the nonlinear shear modulus $\tilde{\mu }(a)=c_{1}+c_{2}/a$, because *g*(*z*) and $\tilde{\mu }(a)$ have different arguments, the curves (*z*,*g*(*z*)) and $\left(a,\tilde{\mu }(a)\right)$ will not coincide in general.

**Figure 5.**
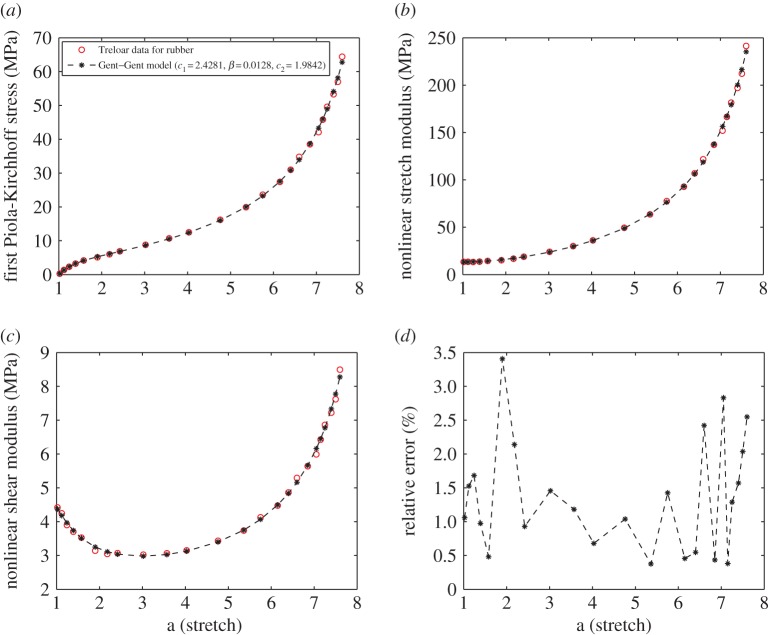
Experimental values for (*a*) the first Piola-Kirchhoff stress *P*(*a*) of rubber in uniaxial tension [52,115], with associated (*b*) nonlinear stretch modulus *E*(*a*) and (*c*) nonlinear shear modulus $\tilde{\mu }(a)$ (table 6), and the corresponding values of the Gent–Gent material model with *c*_1_=2.4281, *β*=0.0128, *c*_2_=1.9842 calibrated to the nonlinear shear modulus $\tilde{\mu }(a)$ and (*d*) the associated relative error. (Online version in colour.)

Here, from the Treloar’s experimental data for uniaxial tension [52,115], we first derive the associated values of the nonlinear stretch modulus *E*(*a*) defined by (3.14). Next, using the universal relation (4.26), the corresponding values of the nonlinear shear modulus $\tilde{\mu }(a)$ are obtained (table 6). The Gent–Gent model listed in table 5 is then calibrated to the data values for the nonlinear shear modulus in the usual way, i.e. by employing a nonlinear least squares procedure to find the minimum of the residual between the nonlinear shear modulus and the given data. The results are plotted in figure 5, where the model parameters are *c*_1_=2.4281, *β*=0.0128, *c*_2_=1.9842 and the relative error is less than 3.4% over all available data. These values are similar to those reported in [52].

**Table 6.** Experimental values for the first Piola-Kirchhoff stress *P*(*a*) of rubber in uniaxial tension [52,115], with associated nonlinear stretch modulus *E*(*a*) and nonlinear shear modulus $\tilde{\mu }(a)$ (figure 5).

	1.02	1.12	1.24	1.39	1.58	1.90	2.18	2.42	3.02	3.57	4.03	4.76
*a*	5.36	5.75	6.15	6.40	6.60	6.85	7.05	7.15	7.25	7.40	7.50	7.60
*P*(*a*) (MPa)	0.2600	1.3700	2.3000	3.2300	4.1600	5.1000	6.0000	6.9000	8.8000	10.7000	12.5000	16.2000
	19.9000	23.6000	27.4000	31.0000	34.8000	38.5000	42.1000	45.8000	49.6000	53.3000	57.0000	64.4000
*E*(*a*) (MPa)	3.3922	13.5394	13.2582	13.6339	14.3691	15.0969	16.7838	18.8941	24.0451	30.0173	36.1431	49.4229
	63.5297	77.5783	92.7688	106.8794	121.7125	137.0535	151.9717	166.4724	181.5243	197.0642	212.1690	241.3236
$\tilde{\mu }(a)$ (MPa)	4.4194	4.2440	3.9007	3.7023	3.5271	3.1423	3.0463	3.0677	3.0237	3.0646	3.1499	3.4352
	3.7370	4.1261	4.4745	4.8623	5.2911	5.6380	5.9887	6.4232	6.8594	7.2205	7.6181	8.4930

### (b) Soft tissues

**Figure 6.**
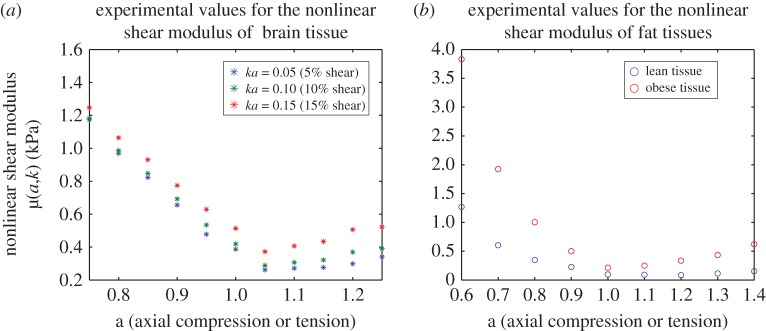
Experimental values for the nonlinear shear modulus *μ*(*a*,*k*) of (*a*) human brain tissue [55] at 5%, 10% and 15% shear strain (table 7) and (*b*) mouse adipose tissues [36] at 3.5% shear strain (table 8), showing a stronger increase under increasing compression than under tension. (Online version in colour.)

Experimental observations on several soft tissues with large lipid content, such as brain, liver and adipose tissues indicate that, under large strains, the nonlinear shear modulus increases strongly and almost linearly as axial compression increases, while increasing only moderately as axial tension increases, regardless of the stress–strain response under simple shear [26,36,55,122] (figure 6 and tables 7–8). Although biological tissues have a viscoelastic mechanical behaviour, hyperelastic modelling is useful as a starting point for the development of more complex models. A hyperelastic constitutive model has a unique stress–strain relationship, which is independent of the strain rate, whereas for viscoelastic materials, the stress–strain response changes with the strain rate. Nevertheless, for some soft tissues where the shape of the stress–strain curve is almost invariant with respect to strain rate, at fixed strain rate, the shear modulus may be captured by a nonlinear hyperelastic model. For human brain tissue, in [55], Ogden-type constitutive models were calibrated, for the first time, to the nonlinear shear modulus *μ*(*a*,*k*) given by (4.9) identified from experimental data collected under multiaxial loading up to 20% shear strain superposed on up to 25% of axial tension or compression. Similarly, for mouse brain and adipose tissues, in [36], hyperelastic models were calibrated to experimental data measuring the nonlinear shear modulus $\tilde{\mu }(a)$ given by (4.14) under small shear superposed on up to 45% axial tension or compression. Currently, experimental data on soft tissues under multiaxial loading are rare, maybe also because they are harder to analyse. The nonlinear shear modulus can be a useful in quantifying results from such experiments.

**Table 7.** Experimental values for the nonlinear shear modulus *μ*(*a*,*k*) of human brain tissue [55] at 5%, 10% and 15% shear strain (figure 6*a*).

*a*	0.75	0.80	0.85	0.90	0.95	1	1.05	1.10	1.15	1.20	1.25
brain tissue											
*μ*(*a*,*k*) (kPa)	1.1738	0.9689	0.8228	0.6560	0.4782	0.3876	0.2619	0.2722	0.2768	0.2987	0.3405
*ka*=0.05											
*μ*(*a*,*k*) (kPa)	1.1817	0.9867	0.8474	0.6928	0.5339	0.4192	0.2888	0.3073	0.3213	0.3696	0.3913
*ka*=0.10											
*μ*(*a*,*k*) (kPa)	1.2478	1.0643	0.9309	0.7744	0.6297	0.5135	0.3723	0.4068	0.4341	0.5064	0.5221
*ka*=0.15

**Table 8.** Experimental values for the nonlinear shear modulus *μ*(*a*,*k*) of mouse adipose tissues [36] at 3.5% shear strain (figure 6*b*).

*a*	0.6	0.7	0.8	0.9	1	1.1	1.2	1.3	1.4
*ka*=0.035									
*μ*(*a*,*k*) (kPa)	1.2687	0.6038	0.3498	0.2272	0.0969	0.0911	0.0846	0.1144	0.1539
lean tissue									
*μ*(*a*,*k*) (kPa)	3.8295	1.9238	1.0036	0.4999	0.2142	0.2494	0.3363	0.4340	0.6205
obese tissue									

### (c) Foams

Solid cellular bodies, or foams, are ubiquitous in nature and engineering applications, and can be found in both load- and non-load-bearing structures [8,123–127]. For soft cellular structures with components exhibiting material nonlinear elasticity, bridging the microstructural responses of individual cells with the apparent macrostructural behaviour is a challenging modelling problem in materials science. To date, there are no established continuum models for this type of structures, even though, in principle, they should stand on the shoulders of the existing nonlinear elasticity theory.

The Blatz-Ko model [83,128] is a phenomenological extension to hyperelasticity of the isotropic linearly elastic models for stretch-dominated structures due to Gent & Thomas [129,130]. The Hill-Storakers foam model extends the Ogden-type strain-energy function for incompressible materials [102] to the compressible case. In [131], it was noted that Hill’s strain-energy function [132] can be used to describe the simple special case of foams where the principal stresses are uncoupled, i.e. depend only upon the stretch ratio in the corresponding principal direction. For these models, in table 9, we write explicitly the nonlinear shear modulus *μ*(*a*,*k*) defined by (4.9), its limit in the case of small shear superposed on finite axial stretch, $\tilde{\mu }(a)=\lim_{k\to 0}\mu (a,k),$ given by (4.14), and its linear elastic limit, $\bar{\mu }=\lim_{a\to 1}\lim_{k\to 0}\mu (a,k)=\lim_{a\to 1}\tilde{\mu }(a),$ given by (4.16), as well as the Poisson function *ν*^(H)^(*a*)=*ν*_0_(*a*) defined by (3.7) and its linear elastic limit $\bar{\nu }=\lim_{a\to 1}\nu^{(H)}(a)$ given by (3.8). The corresponding nonlinear shear modulus under simple shear, $\hat{\mu }(k)=\lim_{a\to 1}\mu (a,k),$ defined by (4.13), can also be derived, and as the Poisson’s ratio is independent of deformation, i.e. $\nu^{(H)}(a)=\nu_{0}(a)=\bar{\nu }$, the nonlinear stretch modulus *E*(*a*) can be obtained from the universal relation (4.25).

**Table 9.** Explicit forms of the shear moduli *μ*(*a*,*k*) of (4.9), $\tilde{\mu }(a)=\lim_{k\to 0}\mu (a,k)$ of (4.14), and $\bar{\mu }=\lim_{a\to 1}\tilde{\mu }(a)$ of (4.16), and of the Poisson’s ratios *ν*^(H)^(*a*)=*ν*_0_(*a*) of (3.7) and $\bar{\nu }=\lim_{a\to 1}\nu^{(H)}(a)$ of (3.8) for selected isotropic hyperelastic foam models. For the shear moduli of these compressible materials, the principal stretches are given by (4.4).

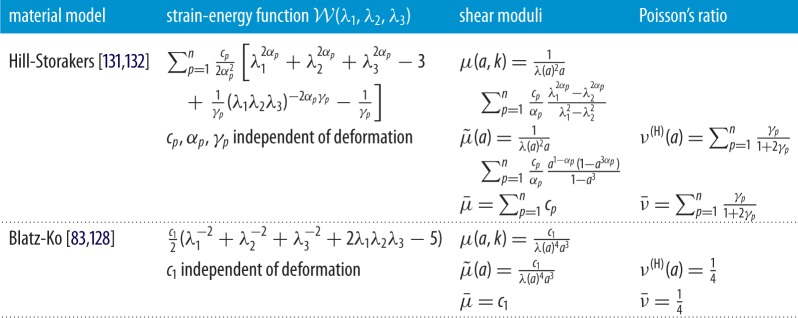

For stretch-dominated foams with arbitrarily oriented cell walls made from a general isotropic hyperelastic material, in [133,134], isotropic hyperelastic models were derived analytically from the microstructural architecture and the physical properties at the cell level. Stretch-dominated architectures, although not load-bearing in general, are structurally more efficient, due to a higher stiffness-to-weight ratio than the bending-dominated ones [8,135–138]. For these models, the nonlinear stretch and shear moduli and the Poisson function can be predicted explicitly from the strain-energy function and the large strains of the cell walls. To illustrate this, we derive here the Poisson function for open-cell foams, and refer to the original papers for further details on how the other elastic parameters may be obtained. For the elastic foams, the geometric assumption is that the cell walls are equal, arbitrarily oriented circular cylinders (or cuboids), with the width to length ratio *ρ*, and the joints between adjacent walls are spheres (or cuboids), with the width much smaller than the length of the walls. The kinematic assumption is that, when the foam is subject to a triaxial stretch, every cell wall deforms by a triaxial stretch, without bending or buckling, and the stretches of the foam and of the walls are related by a rotation, while the joints do not deform significantly. In particular, we assume that the foam deforms by diag(*α*_1_,*α*_2_,*α*_3_), such that *α*_1_=*α*_2_, in the Cartesian directions (**e**_1_,**e**_2_,**e**_3_), with some of the cell walls deforming by diag(λ_1_,λ_2_,λ_3_), such that λ_1_=λ_2_, in the orthonormal directions (**n**_1_,**n**_2_,**n**_3_), given by Mihai *et al*. [133]

6.1 $$\begin{array}{rl} {\text{n}}_{1} & =-{\text{e}}_{1}\;\cos\theta \cos\phi -{\text{e}}_{2}\;\cos\theta \sin\phi +{\text{e}}_{3}\;\sin\theta ,\\ {\text{n}}_{2} & ={\text{e}}_{1}\;\sin\phi -{\text{e}}_{2}\;\cos\phi \\ \text{and}\;{\text{n}}_{3} & ={\text{e}}_{1}\;\sin\theta \cos\phi +{\text{e}}_{2}\;\sin\theta \;\sin\phi +{\text{e}}_{3}\;\cos\theta . \end{array}\}$$

Then the logarithmic Poisson functions (3.7) for the foam and the cell wall are, respectively,

6.2 $$\nu_{0}^{\left(f\right)}=-\frac{\ln\;\alpha_{1}}{\ln\;\alpha_{3}}\;\mathrm{and}\;\nu_{0}=-\frac{\ln\;\lambda_{1}}{\ln\;\lambda_{3}}.$$

In general, the stretches of the cell walls and of the foam are related by Mihai *et al*. [133]

6.3 $$\begin{array}{rl} \frac{\lambda_{1}+\rho }{1+\rho } & =\alpha_{1}\;\cos^{2}\;\theta \;\cos^{2}\;\phi +\alpha_{2}\;\cos^{2}\;\theta \;\sin^{2}\;\phi +\alpha_{3}\;\sin^{2}\;\theta ,\\ \frac{\lambda_{2}+\rho }{1+\rho } & =\alpha_{1}\;\sin^{2}\;\phi +\alpha_{2}\;\cos^{2}\;\phi \\ \text{and}\;\frac{\lambda_{3}+\rho }{1+\rho } & =\alpha_{1}\;\sin^{2}\;\theta \;\cos^{2}\;\phi +\alpha_{2}\;\sin^{2}\;\theta \;\sin^{2}\;\phi +\alpha_{3}\;\cos^{2}\;\theta , \end{array}\}$$

hence if *α*_1_=*α*_2_ and λ_1_=λ_2_, then *α*_*i*_=(λ_*i*_+*ρ*)/(1+*ρ*), *i*=1,2,3. In this case, defining λ_3_=*a*, we obtain the following relation between the Poisson functions for the foam and for the cell wall, given by (6.2):

6.4 $$\nu_{0}^{\left(f\right)}(a)=-\frac{\ln(a^{-\nu_{0}(a)}+\rho )-\ln(1+\rho )}{\ln(a+\rho )-\ln(1+\rho )}.$$

In the linear elastic limit, ${\bar{\nu }}^{\left(f\right)}=\lim_{a\to 1}\nu_{0}^{\left(f\right)}(a)=\lim_{a\to 1}\nu_{0}(a)=\bar{\nu }$, i.e. the respective Poisson’s ratios coincide [133]. Note that, in general, when the Poisson function of the cell wall material is constant, i.e. $\nu_{0}(a)=\bar{\nu }$, the Poisson’s ratio of the foam given by (6.4) is not.

## 7. Conclusion

Constant material parameters are standard in engineering applications where linear elastic models are commonly used. In nonlinear elasticity, similar constitutive parameters can be defined that are functions of the deformation. In this review, we present in a unified mathematical framework several of these parameters, including the stretch modulus, the shear modulus and the Poisson function, which are defined for compressible and incompressible homogeneous isotropic hyperelastic materials and are measurable under axial or shear experimental tests. These functions are important because they represent changes in the material properties as the deformation progresses, and can be identified with their linear elastic equivalent when the deformations are small (tables 1–3). The universal relations between these parameters given in table 4 can be used to quantify nonlinear elastic responses in hyperelastic models.

The nonlinear parameters identified here play significant roles in both the fundamental understanding and the application of many elastic materials under large elastic strains. As shown by our microstructure-based foam models, they can also provide a flexible basis for the coupling of elastic responses in multi-scale processes, where an open challenge is the transfer of meaningful information between scales. Similar parameters can be identified for homogeneous anisotropic elastic materials, where different constitutive parameters may be found in different directions.

## Data accessibility

The datasets supporting this article have been included in the main text.

## Appendix A. Principal stresses

In this appendix, we include several equivalent forms for the principal components of the stress tensors given in §2b. For compressible hyperelastic materials, the principal components of the stress tensors (2.3), (2.7) and (2.8) are defined as follows:

— If {λ_*i*_}_*i*=1,2,3_ are the principal stretches of the given deformation, then the principal components of the Cauchy stress tensor (2.3) are [64, p. 143]
  A 1 $$\sigma_{i}=J^{-1}\frac{\partial W}{\partial \lambda_{i}}\lambda_{i}=J^{-1}\frac{\partial W}{\partial (\ln\;\lambda_{i})},\;i=1,2,3,$$
  or equivalently, by the representation (2.4),
  A 2 $$\sigma_{i}=\beta_{0}+\beta_{1}\lambda_{i}^{2}+\beta_{-1}\lambda_{i}^{-2},\;i=1,2,3.$$
  The principal Cauchy stresses (A 1) are also equivalent to
  A 3 $$\sigma_{i}=\frac{\partial W}{\partial \iota_{1}}\frac{\partial \iota_{1}}{\partial \ln\;\lambda_{i}}+\frac{\partial W}{\partial \iota_{2}}\frac{\partial \iota_{2}}{\partial \ln\;\lambda_{i}}+\frac{\partial W}{\partial \iota_{3}}\frac{\partial \iota_{3}}{\partial \ln\;\lambda_{i}}=\zeta_{0}+\zeta_{1}\ln\;\lambda_{i}+\zeta_{-1}{\left(\ln\;\lambda_{i}\right)}^{-1},\;i=1,2,3,$$
  where
  A 4 $$\begin{array}{rl} \iota_{1} & =\ln\;\lambda_{1}+\ln\;\lambda_{2}+\ln\;\lambda_{3},\\ \iota_{2} & =\ln\;\lambda_{1}\;\ln\;\lambda_{2}+\ln\;\lambda_{2}\;\ln\;\lambda_{3}+\ln\;\lambda_{3}\;\ln\;\lambda_{1}\\ \text{and}\;\iota_{3} & =\ln\;\lambda_{1}\;\ln\;\lambda_{2}\;\ln\;\lambda_{3} \end{array}\}$$
  are the principal invariants of the logarithmic stretch tensors ln U and ln V, and
  A 5 $$\zeta_{0}=\frac{\partial W}{\partial \iota_{1}}+\iota_{1}\frac{\partial W}{\partial \iota_{2}},\;\zeta_{1}=-\frac{\partial W}{\partial \iota_{2}}\;\mathrm{and}\;\zeta_{-1}=\iota_{3}\frac{\partial W}{\partial \iota_{3}}$$
  are scalar functions of the invariants (A 4).
— For the first Piola-Kirchhoff stress tensor (2.7), the principal components are
  A 6 $$P_{i}=J\sigma_{i}\lambda_{i}^{-1}=\frac{\partial W}{\partial \lambda_{i}},\;i=1,2,3.$$
  Equivalently,
  A 7 $$P_{i}=\frac{\partial W}{\partial i_{1}}\frac{\partial i_{1}}{\partial \lambda_{i}}+\frac{\partial W}{\partial i_{2}}\frac{\partial i_{2}}{\partial \lambda_{i}}+\frac{\partial W}{\partial i_{3}}\frac{\partial i_{3}}{\partial \lambda_{i}}=\rho_{0}+\rho_{1}\lambda_{i}+\rho_{-1}\lambda_{i}^{-1},\;i=1,2,3,$$
  where
  A 8 $$\begin{array}{rl} i_{1} & =\lambda_{1}+\lambda_{2}+\lambda_{3},\\ i_{2} & =\lambda_{1}\;\lambda_{2}+\lambda_{2}\;\lambda_{3}+\lambda_{3}\;\lambda_{1}\\ \text{and}\;i_{3} & =\lambda_{1}\;\lambda_{2}\;\lambda_{3} \end{array}\}$$
  are the principal invariants of the stretch tensors **U** and **V** and
  A 9 $$\rho_{0}=\frac{\partial W}{\partial i_{1}}+i_{1}\frac{\partial W}{\partial i_{2}},\;\rho_{1}=-\frac{\partial W}{\partial i_{2}}\;\mathrm{and}\;\rho_{-1}=i_{3}\frac{\partial W}{\partial i_{3}}$$
  are scalar functions of the invariants (A 8).
— The principal components of the second Piola-Kirchhoff stress tensor (2.8) are
  A 10 $$S_{i}=\lambda_{i}^{-1}P_{i}=2\frac{\partial W}{\partial \lambda_{i}^{2}},\;i=1,2,3,$$
  or equivalently, by the representation (2.9),
  A 11 $$S_{i}=\gamma_{0}+\gamma_{1}\lambda_{i}^{2}+\gamma_{-1}\lambda_{i}^{-2},\;i=1,2,3.$$
  When the material is incompressible, i.e. $J=\det F=\lambda_{1}\lambda_{2}\lambda_{3}=1$, the principal components of the stress tensors (2.3), (2.7) and (2.8) are given as follows:
— The principal components of the Cauchy stress tensor (2.3) are
  A 12 $$\sigma_{i}=-p+\frac{\partial W}{\partial \lambda_{i}}\lambda_{i}=-p+\frac{\partial W}{\partial (\ln\;\lambda_{i})},\;i=1,2,3,$$
  or equivalently, by the representation (2.6),
  A 13 $$\sigma_{i}=-p+\beta_{1}\lambda_{i}^{2}+\beta_{-1}\lambda_{i}^{-2},\;i=1,2,3,$$
  where *β*_1_ and *β*_−1_ are given by (2.5) and *p* is the arbitrary hydrostatic pressure. The principal Cauchy stresses (A 12) are also equivalent to
  A 14 $$\sigma_{i}=-p+\zeta_{1}\;\ln\;\lambda_{i}+\zeta_{-1}{\left(\ln\;\lambda_{i}\right)}^{-1},\;i=1,2,3,$$
  where *ζ*_1_ and *ζ*_−1_ are given by (A 5).
— For the first Piola-Kirchhoff stress tensor (2.7), the principal components are
  A 15 $$P_{i}=\sigma_{i}\lambda_{i}^{-1}=-p\lambda_{i}^{-1}+\frac{\partial W}{\partial \lambda_{i}},\;i=1,2,3,$$
  or equivalently,
  A 16 $$P_{i}=\rho_{0}+\rho_{1}\lambda_{i}-p_{0}\lambda_{i}^{-1},\;i=1,2,3,$$
  where *ρ*_0_ and *ρ*_1_ are given by (A 9) and *p*_0_ is the arbitrary hydrostatic pressure.
— The principal components of the second Piola-Kirchhoff stress tensor (2.8) are
  A 17 $$S_{i}=\lambda_{i}^{-1}P_{i}=-p\lambda_{i}^{-2}+2\frac{\partial W}{\partial \lambda_{i}^{2}},\;i=1,2,3,$$
  or equivalently, by the representation (2.11),
  A 18 $$S_{i}=\gamma_{0}+\gamma_{1}\;\lambda_{i}^{2}-p_{0}\lambda_{i}^{-2},\;i=1,2,3,$$
  where *γ*_0_ and *γ*_1_ are given by (2.10) and *p*_0_ is the arbitrary hydrostatic pressure.
